# Traumatic stress symptoms, mental splitting and burnout in health care professionals: a cross-sectional study

**DOI:** 10.3389/fpsyt.2024.1332900

**Published:** 2024-04-11

**Authors:** Norbert Riethof, Petr Bob

**Affiliations:** ^1^ Center for Neuropsychiatric Research of Traumatic Stress, Department of Psychiatry & UHSL, First Faculty of Medicine, Charles University, Prague, Czechia; ^2^ Department of Psychiatry, Faculty of Medicine Pilsen, Charles University, Prague, Czechia

**Keywords:** stress, traumatic stress, burnout, mental splitting, dissociation

## Abstract

**Background:**

Burnout syndrome usually begins with feelings of enthusiasm and idealized visualizations, and it is in contrast with subsequent disillusionment, disappointment, and symptoms which are related to chronic stress experienced later. This tendency to idealization is a parallel to the concept of “mental splitting” described by Kernberg with a pronounced “black and white” perceptual dichotomy between the early idealization and later disillusionment. This study intends examination of relationships between burnout syndrome, traumatic stress and Kernberg’s concept of splitting.

**Methods and participants:**

In this study we have assessed 90 health care professionals (50 women and 40 men) working with a population of diabetic patients utilizing Burnout Measure (BM), Splitting index (SI) and Traumatic Stress Checklist – 40 (TSC-40).

**Results:**

Study results indicate significant Spearman correlations between burnout syndrome (BM) and traumatic stress (TSC-40) in population of men (R=0.75, p<0.01) and of women (R=0.61, p<0.01), as well as between burnout syndrome (BM) and splitting (SI) for both genders: men (R=0.40, p<0.01), women (R=0.51, p<0.01). These findings may have implications for prevention and treatment of burnout syndrome.

**Conclusion:**

The current study findings provide implications that the defensive mechanisms of splitting and traumatic stress may allow for the prediction of burnout symptoms. This relation may potentially be of use in both the potential detection and prevention of burnout syndrome.

## Introduction

There is an ongoing debate what is the relationship between traumatic stress and burnout syndrome. Several research studies indicate the potential of stress as both predictor and accelerant of burnout making burnout syndrome potentially classified as mental disorder (1–5). It appears that the symptomatology of stress and burnout syndrome exhibit similar ‘qualitative’ characteristics, especially in the early stages of burnout (1, 2, 6, 7). These stress influences, and especially traumatic stress related to a traumatic event significantly affect mental stability and subjectively experienced meaning of life, which may accelerate burnout progression (2, 8).

In addition, stress influences on burnout may be combined with cognitive and affective predispositions manifesting as attitudes of enthusiasm and idealized visualizations in the afflicted individuals (6, 7). During the initial stages of burnout there is a tendency to overload and overcommitment reflecting unrealistic expectations about the given individual’s capabilities, but at the same time neglecting personal needs, and experiencing the “all good”, naïve enthusiasm in the early days of work which is in sharp contrast with disappointment and disillusionment later (6, 7, 9). The propensity toward idealization and a binary “black-and-white” conceptualization exists in parallel to Kernberg’s concept of mental splitting (10) which is characterized by oscillating between contradictory perceptions toward the same object (based on painful-bad-punishing and pleasurable-good-rewarding experiences). In accordance with these tendencies, the experience of a given object’s contradictory qualities (“all good” or “all bad”) is associated with undifferentiated self-object representations (10, 11).

In recent research, there is rare evidence about the specific relationships of stress, mental splitting and symptoms of burnout syndrome. Within this context, the current study aims to examine the relationships between burnout symptoms, splitting and traumatic stress.

## Methods and participants

Study participants included 90 members (diabetologists, medical nurses and other professionals who were regular full time workers in hospitals) of the Czech Diabetes Society, a non-profit organization representing health care professionals in the Czech Republic with an interest in diabetes and related topics. When recruiting for the participation in this research, the study purposes and guidelines were announced to all members of the Czech Diabetes Society (N=820). The potential participants had 2 months of a recruitment period (from October 1 to November 30, 2019) to send their answer if they were interested in participating in this study. Immediately after that a link to online questionnaires were sent to those who were interested to participate in this research. All questionnaires used in this research were administered by all participants via this online platform that was specifically prepared and designed for purposes of this study. In total, 90 participants responded the online questionnaires. This group of participants included 50 women and 40 men (mean age 48.99, SD 8.82, age range 31 – 60 years).

The study design has been arranged according to the STROBE criteria for cross-sectional studies. The subject’s participation in the current study was approved by the Czech Diabetes Society and Charles University Medical psychology ethical board and all recruited participants provided written informed consent. All procedures performed in studies involving human participants were in accordance with the ethical standards of the institutional and/or national research committee and with the 1964 Helsinki declaration and its later amendments or comparable ethical standards.

### Psychometric measures

#### Burnout Measure (BM)

The study participant’s level of burnout was assessed utilizing the Burnout Measure (BM) (12, 13). The original 21-item BM total-score was included for reasons of comparability with other studies and for more generic view on burnout as a mental disorder (rather than other instruments for measuring burnout e.g., MBI). BM-items were scored on a 7-point rating scale ranging from 1 “never” to 7 “always” (14).

#### Splitting Index (SI)

Symptoms of splitting were evaluated utilizing a self-reported Splitting index (SI) (11) which has been proposed to test defense mechanisms as described by Otto Kernberg (10). Splitting Index is 24-items self-reported questionnaire utilizing a 5-point Likert scale. Factor analysis differentiates three clusters of items which are identified to enable description of the splitting process. These three identified factor clusters represent the self-factor (splitting of the self-image), the family factor (splitting of images of family members), and the factor of others pertaining to people outside the family.

#### Trauma Symptoms Checklist (TSC-40)

Traumatic stress symptoms were evaluated utilizing the Trauma Symptoms Checklist (14). TSC-40 is a self-reported questionnaire with 40 items scored on a 4-point Likert scale (total score from 0 to 120). TSC-40 assesses stress symptoms in adult individuals associated with childhood or adult traumatic experiences and measures aspects of posttraumatic stress and other symptom clusters found in some traumatized individuals. The TSC-40 measure includes subscales for dissociation, anxiety, depression, sexual abuse trauma index, sleep disturbances and sexual problems. The Czech version of the TSC-40 has high reliability and internal consistency.

### Statistical methods

Statistical evaluations of psychometric measures included means, standard deviations, Spearman correlation. All the methods of statistical evaluation were performed using the software package Statistica version 6. Because the data did not have normal distribution, we have used non-parametric statistical analysis using Spearman correlations coefficients. The main advantage to use non-parametric analysis is its very conservative approach to outliers and leverage points which in the case of using parametric correlations or regression analysis may create false results and increase risk of inappropriate rejection of the null hypothesis (15). In addition, as previous research has indicated, this statistical analysis is appropriate for psychopathological data reflecting traumatic stress symptoms that usually does not have normal distribution (16).

## Results

Results (**Table 1**) indicate significant Spearman correlations of burnout (BM) with traumatic stress symptoms (TSC-40) including subscales for dissociation, anxiety, depression, sexual abuse trauma index, sleep disturbances and sexual problems as well as with symptoms of splitting. With exception of the Spearman correlation of TSC-40 subscale for dissociation with burnout (BM) we did not find statistically significant differences between women and men.

**Table 1:** Spearman correlations of burnout (BM) manifestations with splitting, stress related symptoms (TSC-40) and its subscales for dissociative symptoms (Dis), anxiety (Anx), depression (Dep), Sexual Abuse Trauma Index (SATI), Sleep disturbances (Sleep) and Sexual Problems (Sex) in women (W) and men (M).

	SI		TSC-40		TSC-40		TSC-40		TSC-40		TSC-40		TSC-40		TSC-40	
			Total		Dis		Anx		Dep		SATI		Sleep		Sex	
	W	M	W	M	W	M	W	M	W	M	W	M	W	M	W	M
**BM**	0.51	0.40	0.61	0.75	**0.39**	**0.70**	0.47	0.73	0.60	0.68	0.45	0.52	0.48	0.51	0.36	0.47
**SI**	–	–	0.62	0.38	0.43	0.32	0.44	0.42	0.49	0.31	0.56	0.32	0.35	0.17	0.54	0.31
**TSC-40**			–	–	0.78	0.93	0.80	0.85	0.90	0.87	0.83	0.83	0.78	0.79	0.73	0.65
**TSC-Dis**					–	–	0.59	0.83	0.64	0.79	0.74	0.82	0.59	0.77	0.49	0.56
**TSC-Anx**							–	–	0.66	0.66	0.61	0.62	0.54	0.60	0.57	0.41
**TSC-Dep**									–	–	0.68	0.76	0.81	0.83	0.62	0.46
**TSC-SATI**											–	–	0.53	0.63	0.75	0.70
**TSC-Sleep**													–	–	0.33	0.31

## Discussion

The results support the hypothesis assessed in this research study and indicate that symptoms of burnout are related to traumatic stress symptoms and unstable perceptual and emotional patterns related to “splitting” (10, 11). A continuing debate exists as to the relation between traumatic stress and burnout syndrome. Contention exists as to the possibility that both conceptualizations describe the same disorder, which would make burnout syndrome rightly classified as psychopathology (1, 2, 5, 7, 17).

Previous research suggests that the two characterizations burnout and stress are in fact disparate entities, noting as a primary differentiation an association of work or occupation in burnout (burnout syndrome as a work–related disorder), while stress reactions also exist outside the work-related context (3). However, the findings are mixed with some studies asserting that burnout and stress are not solely independent (4). Burnout might be considered as a stress disorder because stress is a central component of burnout syndrome. There is no burnout without stress (7). Stress from work-related activities seems to be present at least at the beginning of burnout syndrome development in each burnout case (1, 2, 6, 7). On the other hand, there are studies suggesting that stress is not the main cause of burnout, and that job stress alone does not cause burnout, although it can accelerate its evolution (2, 5, 8).

In this connection, results of this study indicate that burnout is statistically related to chronic stress symptoms reflecting individual ontogenesis which is significantly influenced by experienced traumatic events such as abuse or neglect and sexual violence and abuse (16, 18). In addition, results of this study indicate significant relationship of burnout with dissociative symptoms reflecting very serious stressful events which may influence disintegration of conscious experience (16, 19).

Furthermore, the results show significant relationship between burnout and symptoms of splitting. Experienced traumatic stress may also induce splitting as a specific form of dissociation which reflects shifts of mind related to a consciously experienced conflict of opposing mental forces (10, 11). This fragmentation of conscious experience represented by unexpected shifts between devaluation and idealization of other persons and the self is typically related to acute or long-term stressful experiences that also play a significant role in etiopathogenesis of stress disorder (20). In agreement with previous findings the present study indicates relationships of burnout with depression and anxiety (1, 2, 5, 21–26).

The relationship between traumatic stress and burnout symptoms is highly significant and for example according to Chirico (23) the current diagnostic tools are unable to clearly differentiate stress disorders from burnout, particularly in the early stages of the burnout syndrome process when the symptoms are very similar (5, 17).

Main limitation of this study with respect to its conclusions and implications is the cross sectional design which does not allow to make statements about causality of the studied variables and using self-reported measures. Certain role may play unrecognized confounding factors represented by possible variables that were not assessed in this study such as specific work load differences and working conditions among the participants. Nevertheless further research involving larger sample is necessary for the generalizability of the current results.

## Conclusion

Findings of this study suggest that the defense mechanism related to traumatic stress such as mental splitting, or dissociation might predict development of burnout and could be used in screening and prevention programs of burnout syndrome.

## Data availability statement

The raw data supporting the conclusions of this article will be made available by the authors, without undue reservation.

## Ethics statement

The studies involving humans were approved by Charles University, First Faculty of Medicine Ethical Board. The studies were conducted in accordance with the local legislation and institutional requirements. The participants provided their written informed consent to participate in this study.

## Author contributions

NR: Conceptualization, Data curation, Funding acquisition, Investigation, Methodology, Project administration, Writing – original draft. PB: Conceptualization, Data curation, Formal analysis, Funding acquisition, Investigation, Methodology, Project administration, Writing – original draft, Writing – review & editing.
